# A descriptive study of the health information needs of Kenyan women in the first 6 weeks postpartum

**DOI:** 10.1186/s12884-017-1576-1

**Published:** 2017-11-16

**Authors:** Everlyne Rotich, Liz Wolvaardt

**Affiliations:** 1 School of Nursing, Moi University, P.O Box 3900-30100, Uasin Gishu County, Eldoret, Kenya; 2 School of Health Systems and Public Health, University of Pretoria, Private Bag X323, Pretoria, 0001 South Africa

**Keywords:** Health information, Mother-baby pair, Postnatal care, Postpartum needs

## Abstract

**Background:**

A large number of maternal and neonatal deaths occur during birth and 48 h after birth. The benefits of postnatal care to the mother and newborn cannot be overemphasized as this is another opportunity where complications that might arise from pregnancy and childbirth can be treated, as well as the time to provide important information on maternal and newborn care after delivery. This study aimed to determine the information needs of mother-baby pairs in the first 6 weeks after birth.

**Methods:**

An exploratory qualitative study using in-depth interviews at three points in time was conducted with 15 women who had their births at Moi Teaching and Referral Hospital, Kenya. The first interview was done within 48 h after birth followed by a telephone interview at 2 weeks and at 6 weeks after birth. Data were audio recorded and transcribed. Transcripts and field notes were analyzed using thematic content analysis and NVIVO 11 software. Ethical approval was obtained before commencement of studies and permission to conduct the study granted by the chief executive of the hospital.

**Results:**

The only health needs that participants reported were unmet health information needs. Four major themes emerged from the study*.* ‘Connecting with baby’ centered on understanding and meeting baby’s needs, monitoring growth and progress and protecting the baby. The second theme: “Birth as a unique encounter’ is a blend of what was found to be new and a unique. The theme ‘Regaining self’ is a combination of managing self as a mother and handling discomfort related to birth. The final theme: ‘Disconnected information’ is a collection of unmet information needs, the need for clarity in information booklets and conflicting information by different providers.

**Conclusions:**

Participants used the hospital stay as an opportunity to receive more detailed information on how to take care of their babies both directly after birth and in the longer term. Participants had a range of unmet health information needs that extended beyond family planning and fertility. Needs extended to their own care and management of discomforts. The need for consistency in health information by different providers and updated printed material on postnatal care that includes sensitive information and allows opportunities for personalized information was highlighted.

**Electronic supplementary material:**

The online version of this article (10.1186/s12884-017-1576-1) contains supplementary material, which is available to authorized users.

## Background

Postnatal care is defined as care given to women and newborns immediately after birth up to 6 weeks thereafter [1]. The postnatal period is the time that complications arising from pregnancy and birth can be identified, a time that prompt treatment can be provided, as well as a time to promote health within the family. In Kenya more than half of women (53%) do not receive any postnatal care and less than one-fifth receive postnatal care within 41 days after delivery [2]. Low use of postnatal care contributes to high maternal and neonatal mortality. According to the 2008/09 Kenya Demographic and Health Survey, the low uptake of postnatal care in Kenya is positively associated with the mother’s age at delivery of her last child, urban residence, delivery in presence of skilled attendant at birth and attendance of antenatal clinic four times or more [2]. Likewise in other parts in Africa, the factors that have been associated with the low utilization of antenatal and postnatal care are financial, cultural and communication between formal health providers, traditional birth attendants and other community health workers [1]. Sines et al. (2007) found that cultural, educational, social and economic barriers contribute to women not seeking postnatal services [3]. Some of the factors were associated with the low education levels of women, ignorance of women on pregnancy-related complications, high parity, non-use of antenatal care, having birth outside health facilities, birthing with the help of untrained birth attendants and distance to health facilities [3].

Low income countries experience challenges in provision of postnatal care due to lack of a defined postnatal care package [4]. In addition the factors that contribute to low utilization of postnatal services are related to health care provider and facility factors. Health facilities need to be adequately equipped and supported by adequate legal and policy infrastructure [5]. Other factors that contribute to low utilization include unavailability of midwives, inconsistent and prescriptive advice by health care providers, limited information on emotional care and long-term needs of the mother and the baby, information on birth control and health problems [6]. Warren et al. report a deficit on healthcare providers’ knowledge in Kenya on aspects of comprehensive postnatal services and counseling [7]. The study reported a decline in the number of health care providers who asked postnatal women about newborn danger signs at 2 weeks and another significant decline at 6 weeks post-delivery.

Family Care International – a non-profit global health organization that deals with maternal health in different countries – noted a deficit in postnatal care provided in Migori and Homabay, Kenya. In these two rural areas the community perceived that postnatal care only focused on the needs of the newborn leaving out the needs of the mother [8]. They stated that postnatal care mainly dealt with immunization of children. Kenya has a well-established system of universal health services directed at meeting the needs of pregnant women, infants, and young children provided at multiple contact points through the national hospital insurance fund. The system ensures delivery of free maternity care and free universal services for children from birth to the age of 5 years. The health insurance provides rebates for certain services utilized in specified health service delivery plans.

The foundation for health service delivery priorities in Kenya is the Kenya Essential Package for Health, which has six levels for care delivery [9]. These are: level six tertiary (referral and teaching hospitals); level five (secondary hospitals); level four (primary) hospitals; level three health centers, maternities and nursing homes; level two dispensaries and clinics; and level one community level services. Level three to six facilities all offer maternity services, while level two facilities offer mainly antenatal and postnatal services. The main activities of level four to six are curative and rehabilitative, while levels one to three offer promotive, preventive and curative services [9]. Due to this arrangement and midwives being the main providers of maternity care in Kenya, almost all women will receive the care of a midwife at some point during pregnancy, labour and birth and/or the postnatal period.

According to the WHO, postnatal care services should be provided within 48 h, 1–2 weeks and at 6 weeks after birth in order to realize the benefits of postnatal care [10]. Postnatal care lowers the likelihood of neonatal and child mortality by influencing the uptake of breastfeeding thereby reducing the incidence of diarrheal diseases. Continuity of care from antenatal to postnatal care provides an opportunity to counsel women on family planning [11] and ensures that women’s fertility needs are met effectively [12]. The first opportunity for postnatal service is at the hospital for those mothers who have their births in a hospital. This first contact is a crucial opportunity to engage with new mothers about health promotion messages. In Kenya, the ministry of health recommends the WHO three postnatal assessments at the recommended time intervals [13]. Midwives or any healthcare provider attending to a woman at birth is expected to provide postnatal information and give a return date as per the recommendation. The objective of this study was to explore the health information needs of mother-baby pairs in the first 6 weeks after birth.

## Methods

### Design and setting

The study was an exploratory qualitative study that used a longitudinal design whereby women were interviewed in three points in time. The study participants were purposively recruited after birth at Moi Teaching and Referral Hospital in Eldoret in Western Kenya which is the second largest referral hospital in the country. The hospital registers approximately 12,000 births per year and the range of stay for mothers who have delivered is 2 to 4 days. The hospital serves populations that span from higher to lower socio-economic status. Data was collected from November to December 2013.

### Participants

Fifteen postnatal women who had their babies at the study site participated in the study. Women with different characteristics: age, parity, mode of delivery at the postnatal unit were purposively approached and requested to participate in the study to achieve maximum sample variability. The inclusion criterion for participation was having delivered at the facility in the past 48 h.

### Data collection

Participants were interviewed at three points in time: within 48 h of birth, 2 weeks and 6 weeks after birth. The first face to face interview at the hospital was done by a research assistant under the supervision of the first author, ER. The interviews at two and 6 weeks were done by telephone by ER. Three of the original participants could not be reached by phone for the follow-up interviews and one mother lost her baby and withdrew from the study after the first interview. Interviewer reliability was achieved through use of standardized questions in the three time periods of data collection. ER also trained and supervised the first four interviews done by the research assistant.

Participants were given a questionnaire to input their demographic data including age, parity, level of education, marital status and address. An interview guide was used during the interviews. The interview guide was based on a literature search and the authors’ personal experience. The interviews were conducted in English and Kiswahili with six exploratory questions and further probing lines of questioning [Additional file 1]:

1. What health information would you like to know about yourself in the next 6 weeks after birth?
2. What health information would you like to know about the baby in the next 6 weeks?
3. What health problems do women experience after birth?
4. What health problems do babies experience in the first 6 weeks of birth?
5. How have your health needs and those of the baby been met during your encounter with a healthcare provider after delivery?
6. Are there any health challenges that you and your baby have experienced after birth?
7. What do you like about your experience at the hospital?

All face to face interviews were audio-recorded by the research assistant. The research assistant was trained by ER on how to ask the questions and how to probe for understanding. The interviews were guided by the interview guide but the order in which questions were asked varied according to participants’ responses. Data cleaning was done after verbatim transcription and responses were organized according to the original order of the questions. The average interview time ranged between 25 and 37 min. Interview times varied as participants were provided an opportunity to respond freely and to exhaust their ideas. Field notes were taken by both ER and the research assistant during data collection, detailing the environment surrounding the interviews, factual events, individuals’ reactions and personal impressions which provided insight during data analysis and writing of the report. Credibility of the study was ensured through contact with participants at three points in time and achieving saturation of data.. No new data was obtained from the participants from the telephone interviews.

### Data analysis

Simple descriptive statistics are used to present demographic characteristics of participants. Data collection and analysis was carried out concurrently. Transcriptions were done by an independent person who was not involved in the study and checked by ER. ER checked the transcriptions for accuracy by listening to the tapes and checking the transcribed text. Data were analyzed using thematic analysis [14] which involved preparation of the transcript, coding of words or sentences relevant to the topic, draft themes generated from the codes in an iterative process, main categories and sub-categories were identified, draft themes reviewed by ER and LW and finally results were written up.

Analysis was done with assistance of NVIVO 11 software for qualitative data analysis. Analysis involved open coding of the interview transcripts. Each transcript was read and re-read to understand the general concepts and data was aggregated into categories, subthemes and themes after which significant in vivo quotes pertaining to the themes were extracted. Coding of data was done by ER and confirmed by LW. Both researchers reached consensus on the themes and subthemes that arose from coding of the data.

### Quality criteria

The credibility of the study was ensured through the 6 weeks engagement with the study participants while transferability was ensured through thick description of the findings. Dependability was ensured as data collection continued until data saturation and iterative data collection and analysis over the 6 weeks. Finally confirmability was ensured by keeping an audit trail and the discussion of the findings with peer experts.

### Ethical considerations

Ethical approval was granted by Moi Teaching and Referral Hospital and Moi University institutional research and ethics committee. Permission to collect data was granted by the hospital chief executive officer. Participation was voluntary and written informed consent was obtained. Participants were given an information sheet detailing the purpose of the study. Participants gave consent for recording their interviews and they were advised that they could withdraw from the study at any time including stopping the interview when they wanted to. Informed consent was translated in Kiswahili language for participants who were not fluent in English. No names are indicated in the data and cannot be linked to an individual participant. Identifying codes e.g. P1 are used in the text to ensure anonymity.

## Results

### Characteristic of study participants

Fifteen women, aged 18 to 30 years, participated in the study. Their parity ranged from 1 to 4. The majority of the participants were married and had obtained secondary and tertiary education (Table 1).

**Table 1:** Participants’ demographic characteristics (*n* = 15)

Characteristics	Range	*n* = 15	Percentage
Age (years)	18	1	6.7
	19–20	4	26.6
	21–25	7	46.7
	26–30	3	20
Parity	1	5	33.3
	2	7	46.7
	3	2	13.3
	4	1	6.7
Level of education	None	0	0
	Primary	3	20
	Secondary	8	53
	Tertiary	4	27
Marital status	Married	12	80
	Single	3	20

Participants did not report any health needs apart from unmet health information needs. Four themes emerged from the data: Connecting with baby, Birth as a unique encounter, Regaining self and Disconnected information (Table 2).

**Table 2:** Themes, subthemes and number of respondents

Themes and subthemes	Number of respondents
*Theme 1: Connecting with baby*	
• Understanding and meeting baby’s needs	16
• Monitoring growth and progress of baby	8
• Protecting baby	7
*Theme 2: Birth as a unique encounter*	
• Something new and unique	5
*Theme 3: Regaining self*	
• Managing self as a mother	6
• Handing discomforts related to birth	9
*Theme 4: Disconnected information*	
• Unmet information needs	4
• Need for clarity in information booklets	3
• Conflicting information by different providers	3

### Theme 1: Connecting with baby

- Understanding and meeting baby’s needs

Participants wanted information with regard to breastfeeding and understanding why babies cry. A particular concern with initiating breastfeeding was information on how to latch and breastfeed the baby ‘*Breastfeeding is a problem… I would like to know how to breastfeed well’* (P15)*.* They also wanted information on how to handle challenges related to breastfeeding: ‘*What do you do with a baby who does not want to be breastfed?’* (P7). Even within the first 48 h participants wanted information on when they should wean the babies and what food to start with: *‘I would like to know the kind of diet the baby should be given…and the way the baby is growing’* (P1)*.* Participants wanted information of the meaning of different expressions and thought babies cried due to being uncomfortable and wanted to know the reason and meaning of each cry: ‘*now see baby is crying… I don’t understand why he is crying’* (P14). Feelings of frustration were expressed by some of the participants for not being able to elicit the reason for the baby’s cry: ‘*How can somebody know that the baby is sick … you don’t know whether it is the stomach, the head…how will you know that the baby is sick?’* (P6).

Participants wanted to be provided with information on how to care for the baby ‘*At least I am interested in the nurse taking care of me to teach me on how to take care of the baby*’ (P12). They expressed interest in being provided with information on how to clean the baby ‘*Just how to take care of the baby in the right way…About cleanliness’* (P11) and information on:‘*how to hold the baby...how to lay the baby…things like those…there are so many things one does not know about*’ (P 1).

Participants also shared their own views on how to care for the baby ‘*before 6 weeks it’s not good for everybody to touch the baby…because somebody can touch before washing their hands bringing germs*’ (P10).

- Monitoring growth and progress of baby

Of importance was information on how to ensure baby is progressing well and on how to monitor growth:: *‘I would like to know how the baby can progress because when he is too small...there is no way of treating him if he gets common cold’* (P15). Also of importance was information regarding the follow-up visits: ‘*My baby! I would like to know his health the way he is progressing*’ (P9).

- Protecting baby

Some participants were concerned that babies are delicate and their bodies are not well developed: ‘*babies have this problem of distress sometimes because the lungs have not developed fully… they also have problems with colic*’ (P3) and *‘why babies suffer from common cold, have rashes and lose weight sometimes’ (P 12).* They wanted information on how to assess the baby and know when they are sick: *‘I would like to be told how to know that the baby is sick and I would like to know diseases babies suffer from’* (P13). Participants were very aware of the baby’s vulnerability to diseases like malaria: *‘So the baby should not sleep outside the net, must use the net because of this malaria’* (P1) and expressed a need for information for less-immediate health concerns: ‘*There are some that have bad teeth…Why is that so*?’ (P6). Participants were interested in knowing how they would protect their babies from getting ill and how they could alleviate any discomforts the baby might experience: *‘I would like to know how to protect the baby from getting those white things in the mouth…Is herbal medicine good?’* (P4).

Participants who had preterm babies or babies with complications had special needs and felt that they needed more attention while at the hospital and at home: *‘for those mothers...mmm…who have given birth prematurely…they should be given counseling’* (P10) *and ‘because I delivered my baby before time, they are supposed to see me twice, I believe there is the routine clinic and a special one for those who have special needs’* (P14).

### Theme 2: Birth as a unique encounter

- Something new and unique

Birth was experienced as mysterious: *‘you know something that you have never experienced before, the first time you have to be shocked’* (P10) with post-delivery experiences that were unexpected: *‘you receive periods for some days...then it stops, then it starts again for a long time’* (P4). Participants who were new mothers experienced birth as mysterious with unexpected post-delivery questions on how to return to normal life: *‘this is something new. After how long will I be allowed to leave the house? You see I have examinations next week and I don’t know whether I can go and do them? Will I be still weak?’* (P2).

### Theme 3: Regaining self

- Managing self as a mother

In managing self as a mother, participants wanted information on what they should do to stimulate their appetite and what kind of food to eat after birth: *‘I have no appetite, I am not able to eat… I need to know the kind of food to eat’* (P8). Some of their dietary questions were prompted by the need to produce enough milk: ‘*They are saying that take water, take water…Take porridge, I’m taking hot things like tea… I am taking until now I don’t know… But I’m worried, is it that because I delivered prematurely or what? That’s what I want to know…I want to know if there’s any injection that somebody can be given for the milk*?’ (P5). Participants also wanted to be provided with information on care of their breasts and when they ‘*should come for check* [up visits]’ (P9).

- Handling discomforts related to birth

Participants wanted information on how to handle discomforts related to childbirth and identify their own healing progress: *‘Like to get the energy when there is dizziness…When is not there, how to progress healthwise?’* (P13). Participants mentioned the discomfort they experienced: *‘you can have headaches…like right now I am still bleeding, I have stomach pains’* (P9) and wanted information on the reasons for certain symptoms: *‘I don’t know whether the blood has reduced…like this dizziness’* (P13) as well as possible complications after discharge: *‘There are those complications that you cannot know immediately…maybe somebody can go home…maybe the wound will open… how do you prevent that?’* (P12). Those participants who had undergone caesarean section wanted to know how to take care of the wound and information on how long it takes for the wound to heal and what kind of activities to engage in: *‘when the scar will heal…then the work that I will not be doing because operation is not a normal thing. It’s not like the other deliveries’* (P10).

### Theme 4: Disconnected information

- Unmet information needs

Although participants received information on family planning, they stated that the information was not adequate as they wanted to know more: ‘ *mmm, they give us half half information. Like the period you are supposed to protect yourself from getting pregnant after birth…they just tell you you will get more information at the clinic’* (P3). Participants wanted information on when to resume sexual relations with their husbands: ‘*they just tell us you will be given more information at the clinic. Then I don’t know what to do…I would like to know how to behave with my husband’* (P4).

Participants wanted to be informed about procedures done on their baby: *‘Sometimes they don’t tell you why they do some things…for example blood was taken from my baby…and I don’t know they were taking the blood for’* (P3). Another aspect of provision of information is the timing of the communication *‘you see I delivered yesterday…I was fatigued…I was defeated to push…my baby took time to cry…is there someone who will direct me?’* (P1).

Some participants also wanted information on the safety of the hospital environment: ‘*when you deliver…you can contract some diseases. I want to know whether I have contracted any disease’* (P6).

Also communication with relatives was considered as problematic as: *‘nothing else they know… they only know that I have gone to theatre…but they don’t know anything else, they don’t know the outcome’* (P12).

- Need for clarity in information booklets

Participants questioned the usefulness of some information in the mother-baby booklets provided during antenatal care visits: ‘*now that booklet says the first bath is after 24 hours. Does it mean we shall wait for 24 hours before we bath the baby...now I delivered at five in the morning will I wait until five in the morning?’* (P1)

- Conflicting information by different providers

Participants expressed concern that they received conflicting information from different health care providers *‘one person will tell you give your baby a certain type of food…another one will tell you not that one…another one will tell you apply something to your baby…now all this information…which one do you follow?’* (P8). The participants thought that community health workers in particular did not provide accurate information.

## Discussion

The only health needs that emerged from this study were health information needs. These health information needs did not change over time and no new information was added via the two telephone interviews once the participants were discharged and at home.

Four main themes emerged from the data: connecting with baby, birth as a unique encounter, regaining self and disconnected information.

The participants had concerns for both the health of the baby and their own health and needed information on both. The concerns for the health of the baby were paramount and were both immediate and long-term. One possible reason for the inclusion of long-term concerns and questions could be the lack of trust in the competency of community health care workers.

Participants felt that they did not receive adequate information on connecting with the babies including practical challenges such as how and when to breastfeed. The findings are consistent with a study done by Hoddinnott and Pill, among low income women that showed provision of information alone was not adequate [15]. The need for information on caring for the baby also concurs with Lugina et al. findings from a study in Tanzania where women were examined in the first and 6 weeks after birth and mothers’ concerns on baby care were on how to breastfeed, hold, bath and prevent the baby from getting infections [16]. This finding is similar to Coates’ study on postnatal distress where mothers were determined to succeed in breastfeeding [17]. In our study, there were also those participants – those with preterm babies – who had special needs and required additional attention both while at hospital and at home.

Participants in this study described birth as a new encounter as they were experiencing different changes after birth and not really understanding the changes in their own bodies. A study done in two rural areas in Kenya was of the view that postnatal care focused on the needs of the newborn leaving out the needs of the mother [8] and in this study both the theme of birth as a new encounter and the theme of regaining self emphasize that participants had health information needs that extended beyond parental care or fertility.

Participants were interested in being provided with information on changes they expect to experience after birth and how to manage post-delivery discomforts and complications. This information need is key as mothers who had been informed of maternal and neonatal complications before discharge are more likely to seek services [18, 19].

The gaps and contradictions in the health promotion communication that participants received are clear. Some of these gaps are possibly due to health care providers not seeing the importance – to them at least – of explaining routine activities such as drawing blood from the babies. Not all participants were aware of what immunizations the babies had received before discharge which is both critical information and an opportunity to guide mothers on the necessity for follow up visits at 2 and 6 weeks. The more personal subjects such as initiating sex after birth were identified as being poorly communicated and inadequate to their needs. This study supports other findings that health care providers provide inconsistent and prescriptive advice with limited information on emotional care and long-term needs of the baby and the mother [8].

### Strengths and limitations

The longitudinal nature of the study provided insight on the health information needs that postnatal mothers experience over the first 6 weeks. The interaction with women at three points in time provides the actual health information needs required for the women in the three critical postnatal checkups periods and provides information regarding information needs that health care providers can address.

It was challenging to interview mothers by telephone due to communication problems and their divided attention. The size of the sample and the qualitative nature of the study limit the generalizability of the findings to other settings. Interviews of all women who had their deliveries were done together. Using a homogenous group of women such as first-time mothers would have yielded specific information for mothers with different characteristics. Mother-baby health information needs of mothers may vary with parity. Two final limitations are that only women who had their births at an urban, tertiary level institution were included and that the majority of the sample had some form of post-primary qualifications. This sample composition therefore limits generalizability of the findings to those with little or no education, who had their births in a rural setting or rural mothers who possibly benefited from support from relatives.

## Conclusions

The foundation of postnatal care services is the first encounter in the hospital and this encounter is the sentinel – and possibly single – opportunity to communicate important health messages to the mothers. Although standard postnatal care involves providing information to the mother on how to take care of herself and the baby, much progress could be made regarding the accuracy and value of the information. This study suggests that the context of the health education and health promotion booklets needs to be evaluated by future (qualitative) studies to ensure the inclusion of topics that women find useful and based on our findings should probably extend beyond aspects such as fertility. Sensitive topics such as initiating sex after delivery could form part of the standard inclusion in the booklet that is given to mothers. Any future study should be extended to include a broad range of Kenyan women.

The standard postnatal care information should be supplemented by a structured opportunity to ask questions that specifically apply to each individual. One possible solution for such a structured opportunity is through delivery of some of the postnatal services in the community. Extending care beyond facilities and into families and the community is also aligned to the critical time after birth when the majority of maternal and neonatal deaths occur. If postnatal care is to be improved in Kenya, then meeting all health information needs of new mothers in the hospital, the clinics and the community should be at the top of the agenda.

## Additional file

Additional file 1: The interview questions. (DOCX 19 kb)

## References

[1] Partnership for Maternal, newborn and child health. Opportunities for Africa’s newborns: practical data, policy and programmatic support for newborn care in Africa. Cape Town, South Africa: Mills Litho. 2006.

[2] Kenya National Bureau of Statistics (KNBS) and ICF Macro (2010). Kenya demographic and health survey 2008–09.

[3] Sines E, Syed U, Wall S, Worley H. Postnatal care: a critical opportunity to save mothers and newborns. Saving newborn lives. Policy perspectives on newborn health. Save the children. Washington: Population Reference Bureau; 2007. p. 1–8. http://www.prb.org/pdf07/SNL_PNCBriefFinal.pdf.

[4] Kerber K, de Graft-Johnson JE, Bhutta ZA, Okong P, Starrs A, Lawn EJ (2007). Continuum of care for maternal, newborn, and child health: slogan to service delivery. Lancet.

[5] de Bernis L, Sherrat DR, AbouZahr C, Van Lerbeghe W (2003). Skilled attendants for pregnancy, childbirth and postnatal care. Brit Med Bull.

[6] Fenwick J, Butt J, Dhaliwal S, Hauck Y, Schmied V (2010). Western Australian Women’s perceptions of the style and quality of midwifery postnatal care in hospital and at home. Women Birth.

[7] Warren C, Mwangi A, Oweya E, Kamunya R, Koskei N (2010). Safeguarding maternal and newborn health: improving the quality of postnatal care in Kenya. Int J Qual Health C.

[8] Family Care International (2005). Care-seeking during pregnancy, delivery and the postpartum period: a study in Homa Bay and Migori districts, Kenya.

[9] Muga R, Kizito P, Mbayah M, et al. Overview of the health system in Kenya. Demog Health Surv. 2005; https://dhsprogram.com/pubs/pdf/spa8/02chapter2.pdf. Accessed 14 July 2017.

[10] The World Health Organization (2007). The partnership for maternal, newborn and child health: ten year strategy. The World Bank.

[11] National Coordinating Agency for Population and Development (NCAPD) [Kenya], Ministry of Medical Services (MOMS) [Kenya], Ministry of Public Health and Sanitation (MOPHS) [Kenya], Kenya National Bureau of Statistics (KNBS) [Kenya], ICF Macro. 2011. Kenya Service Provision Assessment Survey (KSPA) 2010. http://pdf.usaid.gov/pdf_docs/Pnadx233.pdf. Accessed 11 Nov 2015.

[12] Warren C, Phafoli S, Majara B, Tšukulu T (2008). Extending prevention of mother-to-child transmission through postpartum family planning in Lesotho. Frontiers in reproductive health program, final report.

[13] Manual EOC (2006). For health service providers in Kenya.

[14] Liamputtong P, Ezzy D (2005). Qualitative research methods.

[15] Hoddinott P, Pill R (2000). A qualitative study of women’s views about how health professionals communicate about infant feeding. Health Expect.

[16] Lugina HI, Christensson K, Massawe S, Nystrom L, Lindmark G. Change in maternal concerns during the six weeks postpartum period: a study of primiparous mothers in Dar Es Salaam.Tanzania. J Midwifery Wom Heal. 2001;4:2–48. 10.1016/s1526-9523(01)00133-7 11603640

[17] Coates R, Ayers S, de Visser R (2014). Women’s experiences of postnatal distress: a qualitative study. BMC Pregnancy Childbirth.

[18] Izudi J, Amongin D (2015). Use of early postnatal care among postpartum women in eastern Uganda. Int J Gynecol Obstet.

[19] Phiri PW, Rattanapan C, Mongkolchati A (2015). Determinants of postnatal service utilisation among mothers in rural settings of Malawi. Health Soc Care Comm.

