# Supplementary material for: A descriptive study of the health information needs of Kenyan women in the first 6 weeks postpartum
Source: BMC Pregnancy Childbirth. 2017 Nov 16;17:385. doi: 10.1186/s12884-017-1576-1 (PMC5691856; doi:10.1186/s12884-017-1576-1)
Supplement: Additional file 1: — The interview questions. (DOCX 19 kb) [file 12884_2017_1576_MOESM1_ESM.docx]

INTERVIEW SCHEDULE FOR HEALTH INFORMATION NEEDS FOR MOTHER-BABY PAIRS

1. What health information would you like to know about yourself in the next six weeks after birth?
2. What health information would you like to know about the baby in the next six weeks?
3. What health problems do women experience after birth?
4. What health problems do babies experience in the first six weeks of birth?
5. How have your health needs and those of the baby been met during your encounter with a healthcare provider after delivery?
6. Are there any health challenges that you and your baby have experienced after birth?
7. What do you like about your experience at the hospital?
